# Income-related inequalities in health care utilization in Mongolia, 2007/2008–2012

**DOI:** 10.1186/s12939-015-0185-8

**Published:** 2015-07-25

**Authors:** Javkhlanbayar Dorjdagva, Enkhjargal Batbaatar, Bayarsaikhan Dorjsuren, Jussi Kauhanen

**Affiliations:** Department of Health Policy and Management, School of Public Health, Mongolian National University of Medical Sciences, Zorig street, Ulaanbaatar, 14210 Mongolia; Institute of Public Health and Clinical Nutrition, Faculty of Health Sciences, University of Eastern Finland, Kuopio, Finland; Faculty of Economics and Business Sciences, University of Sannio, Benevento, Italy; Department of Health Systems Governance and Financing, World Health Organization, Geneva, Switzerland

**Keywords:** Concentration index, Horizontal inequity, Inequality, Health care, Mongolia

## Abstract

**Background:**

Although health strategies and policies have addressed equitable distribution of health care in Mongolia, few studies have been conducted on this topic. Rapid socio-economic changes have recently occurred; however, there is no evidence as to how horizontal inequity has changed. The aim of this paper is to evaluate income related-inequalities in health care utilizations and their changes between 2007/2008 and 2012 in Mongolia.

**Methods:**

The data used in this study was taken from the nationwide cross-sectional data sets, the Household Socio-Economic Survey, collected in 2007/2008 and 2012 by the National Statistical Office of Mongolia. We employed the Erreygers’ concentration index to measure inequality in health service utilization. Horizontal inequity was estimated by a difference between actual and predicted use of health services using the indirect standardization method.

**Results:**

The results show that the concentration indices for tertiary level, private outpatient and inpatient services were significantly positive, the contrary for family group practice/*soum* hospital outpatient services, in both years. After controlling for need, pro-rich inequity (*p* < 0.01) was observed in the tertiary level, private outpatient, and general inpatient, services in both years. Pro-poor inequity (*p* < 0.01) existed in family group practice/*soum* hospital outpatient services in both years. Degrees of inequity in tertiary level hospital and private hospital outpatient services became more pro-rich, whereas in family group practice/*soum* hospital outpatient services became more pro-poor from 2007/2008 to 2012. Pro-rich inequity in inpatient services remained the same from 2007/2008 to 2012.

**Conclusions:**

Equitable distribution of health care has been well documented in health strategies and policies; however, the degree of inequity in delivery of health services has a tendency to increase in Mongolia. Therefore, there is a need to consider implementation issues of the strategies and refocus on policy prioritizations. It is necessary to strengthen primary health care services, particularly by diminishing obstacles for lower income and higher need groups.

**Electronic supplementary material:**

The online version of this article (doi:10.1186/s12939-015-0185-8) contains supplementary material, which is available to authorized users.

## Background

Ensuring equality in access to health care is a key objective in any viable health policy. During the past two decades, the number of studies on socio-economic inequalities in health and health care utilization has increased significantly [1]. Thus, there is considerable evidence on income-related inequality in health care utilization both in developed and developing countries [2–8].

Income-related inequality occurs when there are differences in the use of health care services across different income groups. However, inequity in health care use is avoidable inequality, and it exists when there are differences in the use of health care after standardization of different needs among the population [9, 10]. Equitable distribution of health care has two dimensions, horizontal equity and vertical equity. Horizontal equity refers to equal treatment for those who have equal needs, whereas vertical equity refers to unequal treatment for those who have unequal needs [2, 10]. In health care, horizontal inequity is frequently measured both in policy and research. Because many countries pay attention to equal distribution of health care–equal treatment for equal medical need regardless of difference of individual characteristics, such as income, race, etc. Additionally, horizontal equity is easily tested and interpreted than vertical equity. On the other hand, vertical equity is difficult in measuring and interpreting, specifically in countries where there are barriers of access to health services [11]. Practically, vertical equity is convenient to be measured when funding e.g. progressivity in financial contribution is the center of concern, while horizontal equity is measured in terms of access to health services based on a key aim of ensuring equitable services for those who have same needs [12].

Evidence-based, integrated social and economic policy and its effective implementation can have positive impacts on tackling health inequality and increasing equitable accessibility [9]. Ensuring greater equity in use of needed health services of good quality with financial protection is a fundamental policy objective for all countries aimed to move towards universal health coverage [13].

As in other countries, improving population health and ensuring income-related equality in health services has been a central issue of the health sector of Mongolia. Before 1990, health care services were financed and delivered on an equal basis by the government of Mongolia [14, 15]. In 1990, there was a peaceful democratic revolution and as a result transition from centrally planned economy to market-based economy occurred. After the socio-economic transition, in 1994, the government introduced a SHI system to alleviate the burden of the state budget; to protect the population financial hardships; and to ensure better quality and equitable health services [14, 16]. Further, in 2005, the Health Sector Strategic Master Plan 2006–2015 was approved, which aimed to “improve the health status of all the people of Mongolia, especially mothers and children, through implementing sector wide approach and providing responsive and equitable pro-poor, client-centred and quality service.” [17]. Based on the master plan, the government introduced the National Strategy on Health Financing for 2010–2014; its main purpose was “to deliver equitable and accessible quality health care services to the population and to protect them from health associated financial risks” [15, 18].

Although health strategies and policies aimed at equitable distribution of health care, their implementation and equity improvements have been comparatively less studied in Mongolia. A few studies focused on accessibility and inequality in health services [19–21]; however, they limited their investigation to either a specific population group or a given geographical area.

There is one nationwide study that evaluates the income-related inequality in health and health services utilization based on the Mongolian Household Socio-Economic Survey (HSES) of 2007/2008 using ADePT software [22]. Tsilaajav and her colleagues found that income-related inequality in health care utilization in Mongolia does exist; in particular while inpatient and outpatient services of secondary and tertiary level public hospitals are concentrated among the rich, the outpatient care in family group practice (FGP) and *soum* (county) hospitals is concentrated among the poor [22]. However, this report does not measure horizontal inequity in health care utilization.

Since the above-mentioned study, the Mongolian economy has grown stably due to the emerging mining sector and GDP growth was reported to be 17.3 and 12.3 % in 2011 and 2012 respectively. Also, the poverty rate dropped from 35.4 to 27.4 % between 2008 and 2012 [23]. At the same time, urbanization increased, and about 45.9 % of the total population was living in the capital in 2012 [23], even though Mongolia is one of the lowest density countries in the world.

However, there is no evidence as to how horizontal inequity changed over time following these rapid socio-economic changes. For this reason, country-specific data and evidence is still needed for policy discussion and formulation in the context of Mongolia. We hypothesized that income-related inequality in health care utilizations has been expanded in Mongolia during the study years.

Health care services in Mongolia are financed by three main sources, which are the central state budget, the social health insurance (SHI), and out-of-pocket payments (OOP) (Table 1) [16, 24]. In comparison with other developing countries, the SHI coverage is relatively high (98.6 % in 2011) in Mongolia. Nonetheless in 2010, the OOP rose to 41 % of the total health expenditure [16, 18]. This fact is one of the urgent issues in Mongolia. Global and regional evidence suggests that there are negative effects on health service access and use when OOP is more than 20 % of the total health expenditure [25]. On the one hand, OOP directly affects income-related inequality in health services’ access because formal or informal fees and payments required create substantial financial barrier for those who need health services. On the other hand, OOP is the main cause for financial burden for those who need and use them. It often leads to increased incidences of household catastrophic health expenditure and impoverishment especially among lower income population groups [26].

**Table 1:** Health expenditure in Mongolia, 2012

Indicators	Value
Total health expenditure (THE) % GDP	6.3
THE per capita in USD (PPP adjusted)	344.9
General government expenditure on health (GGHE) as % of THE	62.8
Private expenditure on health (PvtHE) as % of THE	37.2
GGHE as % of General government expenditure	9.0
Social security funds as % of GGHE	21.2
Out of pocket expenditure as % of THE	34.6
Out of pocket expenditure as % of PvtHE	93.1

Source: National Health Accounts data, http://www.who.int/nha/en/

In 2013, health care services were delivered through 16 specialized hospitals, five regional diagnostic centers, 20 district and *aimag* hospitals, eight district public health centers, six rural general hospitals, 39 *intersoum* hospitals, 228 family health centers, 271 *soum* health centers, 19 village health centers, 31 hospitals for army, railways, and prisons, 197 private inpatient hospitals, and 822 private outpatient clinics [27].

The aim of this paper is to evaluate income related-inequalities in health care utilizations and their changes between 2007/2008 and 2012 in Mongolia.

## Methods

### Data

The data used in this study were adopted from the nationwide cross-sectional data sets, the HSES, collected in 2007/2008 and 2012 by the National Statistical Office of Mongolia. The aim of the survey is to evaluate and monitor the income and expenditure of households, update the basket and weights for consumer price index, and it offers inputs to the national accounts. The survey is conducted every year with three levels of strata as Ulaanbaatar (the capital city), province centers and rural area by covering all 21 provinces and the capital city of Mongolia. The HSESs are based on the standardized questionnaires that reveal information on elements such as demographics, socio-economic indicators, social transfers, health, housing and education, among others. In the HSES 11,172 and 12,811 households were included in 2007/2008 and 2012, respectively. These households consist of 44,510 and 47,908 individuals in total in 2007/2008 and 2012. Our main inclusion criteria was individuals, who were aged 18 and older. Additionally, we excluded individuals who were: i) a household head or any household student members away from home for 11 months or more; ii) anyone else away from home for 6 months or more.

After we applied the inclusion criteria to the data, we removed cases with missing data. We found that there were only 49 and 17 missing data on income in each year and we eliminated them. Accordingly, 27,681 and 30,567 individuals retained in the studies from 2007/2008 to 2012.

### Dependent variables

Measurements of outpatient care utilization were based on whether individuals received outpatient care by visiting any central hospital/clinic, district/*aimag* hospital/clinic, FGP/*soum* hospital as well as private hospital during the past 1 month or not (yes/no). In Mongolia, primary health care services are delivered by FGPs/village health centers in urban areas and by *soum* and *intersoum* hospitals in rural areas. We used the terms FGP and *soum* hospitals; however, FGP and *soum* hospitals were renamed family health centers and *soum* health centers respectively, according to a revision of the health act in 2011. Inpatient service utilization was measured, if any hospitalization occurred in the past 12 months (yes/no).

### Independent variables

The HSES questionnaires in both years elicited wide range of information about household income. In the analysis, only household net monetary income earned by the household members during the reference years was used. We calculated household income on the basis of sources of income, including wage from work, income from self-employment, agricultural income, private income and pension, among others for both years. In the next step, household income per equivalent adult was estimated in accordance with the OECD modified equivalence scale, adopted by the Statistical Office of the European Union, which is “1 to the household head, of 0.5 to each additional adult member and of 0.3 to each child”.

Need variables used in the paper are age, gender and self-reported health. We generated 14 dummy variables based on age and sex (females aged 18–24, 25–34, 35–44, 45–54, 55–64, 65–74, and 75 or older; males aged 18–24, 25–34, 35–44, 45–54, 55–64, 65–74, and 75 or older). Measurement of health variables is based on four questions which were directly asked from individuals: (a) ‘Have you got any disabilities? (yes/no)’; (b) ‘Did you have any health complaints in the past month? (yes/no)’; (c) ‘Did you miss your work, school or daily activities due to the illness in last month? (number of days)’; and (d) ‘Have you got any chronic illnesses? (yes/no)’ which is available only in the 2007/2008 year’s data. Non-need variables are activity status, marital status, education, location, household size and health insurance coverage. Marital status is categorized into married/living together, divorced/separated, widowed and single/never married. Activity status contains employed, herder, self-employed, inactive and unemployed. Household size is a continuous variable. Location included urban and rural areas. Health insurance is based on whether an individual is covered by the social health insurance.

### Measuring inequality

A wide range of measuring techniques are used to measure inequality in health and health care utilization, including simple, regression based, and more advanced techniques [28]. Among them, the concentration index is the most commonly used method to calculate the degree of income-related inequality in health care utilization [29] due to its direct link to the concentration curve, which shows a complete picture of a share of health service by cumulative proportions of population ranked by income [1].

The concentration index indicates the covariance of the health care utilization and the fractional rank of income distribution as:

1 $$ CI=\frac{2}{\mu }{\operatorname{cov}}_w\left({y}_{it},{R}_i^t\right) $$

where *i* is an individual, *y*_*i*_ is the health care utilization, *μ* is the mean of the health care utilization (*y*), *R*_*i*_ is the individual’s fractional rank in the income distribution and *t* is the year. The concentration index represents the concentration curve as a single number by summarizing the inequality weights at different points in the income distribution. The concentration index falls within a range of −1 and +1. If a value of the concentration index is a negative, it indicates that health care utilization is concentrated among the pro-poor. When a positive value index appears, it shows that health care utilization is concentrated among the pro-rich group [1].

The concentration index depends on a mean value of the health variable (health care utilization). Thus, Wagstaff stated that the concentration index has a limitation, which occurs when health care utilization is binary, because as the mean increases, the concentration index shrinks [30]. Consequently, Erreygers introduced the Erreygers’ concentration index (EI) as a solution for the drawback of the standard concentration index [31], and it is more compatible with binary variable and formulated as this:

2 $$ E(h)=\frac{4\upmu}{\left({b}_n-{a}_n\right)}C(h) $$

where *C*(*h*) represents the standard concentration index presented in equation 1. The *μ* is the mean of health care utilization in population. *b*_*n*_ and *a*_*n*_ are the upper and lower bound of health care utilization. This study used EI owing to the variable’s binary nature.

#### Horizontal inequity

In this study, we estimated horizontal inequity to assess avoidable inequity in health service utilization in the population. Apparently, health care utilization differs among and across the populations as regards the income differences because health care needs differ in the population due to, for example age, gender, health status, and this difference is unavoidable. Therefore, in order to assess if health care utilization is equally distributed in the population regard to income distribution, one should control varying need variables. Thus, horizontal inequity is expressed by a difference of actual inequality in the population and need-standardized utilization of health care. In other words, standardization for differences in need explains unavoidable inequity in health care utilization and a difference between the concentration index and unavoidable inequity demonstrates avoidable inequity in health care utilization [1].

#### Need standardization

We used the indirect standardization method to measure horizontal inequity in health care utilization. Owing to the nature of a binary variable, in general, a non-linear estimation is applied. However, studies on health equity, which have used both a linear and non-linear estimation, showed that the results were consistent in both models. Therefore, we used ordinary least square regression (OLS) [1, 32]. First, coefficients of OLS for actual health care use (*y*_*i*_) were obtained by the following formula:

3 $$ {y}_i=\alpha +\beta \ln in{c}_i+{\sum}_k{\gamma}_{\kappa }{\chi}_{\kappa, i}+{\sum}_p{\delta}_p{z}_{p,i}+{\varepsilon}_i $$

where *y*_*i*_ is health care use of individual, In *inc*_*i*_ represents the logarithm of household income per equivalent adult; *χ*_*k*_ is a set of need variables including age, sex and health needs; *z*_*p*_ is a set of non-need variables consisting of location, insurance, activity status, household size, education and marital status; *α*, *β*, *γ*_*κ*_, and *δ*_*p*_ are the parameter vectors, and *ε*_*i*_ is an error term.

Second, based on equation 3, we generated need-predicted values of health care utilization (*ŷ*_*i*_^*x*^) using the parameter vectors (*α*, *β*, *γ*_*κ*_, *δ*_*p*_), individual values of the need variables (*χ*_*κ*,*i*_ ), sample means of the logarithm of household income (In *inc*_*i*_ ), and non-need (*z*_*p*,*i*_) variables. The equation of the need-predicted value is written as:

4 $$ {\widehat{y}}_i^x=\widehat{a}+\widehat{\beta}\mathrm{In} in{c}^m + {\displaystyle \sum_{\kappa }{\widehat{\gamma}}_k{\chi}_{\kappa, i}} + {\displaystyle \sum_P{\widehat{\delta}}_p{z}_p^m} $$

Finally, the estimate of indirectly standardized health care utilization (*ŷ*_*i*_^*IS*^) was simply obtained from the difference between actual (*y*_*i*_) and need-predicted health care utilization (*ŷ*_*i*_^*X*^), and the sample mean (*y*^*m*^) was added [1].

5 $$ {\widehat{y}}_i^{IS}={y}_i - {\widehat{y}}_i^X + {y}^m $$

#### Decomposition analysis

It is evident that how much various factors contribute separately to income-related inequality in health care utilization with the decomposition analysis [1]. There has been argument that decomposition analysis is not developed for a linear regression model and when it is used in a non-linear model for binary outcome, it introduces an approximation error. However, the decomposition analysis only requires using the OLS coefficients, not the predicted values; thus, this is not a problem [3].

Regarding the transformation of health care utilization, the EI is equal to the decomposition of the concentration index multiplied by 4 and *μ*_h_. Thus, the EI for health care utilization can be written as:

6 $$ E=4\left[\beta {\mu}_y{C}_y+{\displaystyle \sum_j}{\gamma}_j{\mu}_{zj}{C}_{zj}+{\displaystyle \sum_k}{\delta}_k{\mu}_{xk}{C}_{x,k}\right] $$

where *μ* represents the mean, *j* and *k* are vectors of variables *z*_*j*_ and *x*_*k*_, *γ* and *δ* represent the coefficient of the variable *z* and *x*, respectively. C represents the concentration index [31].

The main interest of this work was to analyse how horizontal inequity changed between 2007/2008 and 2012; and in order to accomplish that, the Oaxaca decomposition analysis was used [33, 34].

7 $$ \varDelta C={\displaystyle \sum_k}{\eta}_{kt}\left({C}_{kt}-{C}_{kt-1}\right)+{\displaystyle \sum_k}{C}_{kt-1}\left({\eta}_{kt}-{\eta}_{kt-1}\right)+\varDelta \left(G{C}_{et/\ }{\mu}_t\right) $$

An alternative of the Oaxaca decomposition analysis can be written as:

8 $$ \varDelta C={\displaystyle \sum_k}{\eta}_{kt-1}\left({C}_{kt}-{C}_{kt-1}\right)+{\displaystyle \sum_k}{C}_{kt}\left({\eta}_{kt}-{\eta}_{kt-1}\right)+\varDelta \left(G{C}_{et/\ }{\mu}_t\right) $$

where *η*_*kt*_ represents the elasticity of variable *k*, *t* is the year, and Δ denotes differences. The Oaxaca decomposition allows to show changes in income-related inequality in health care use as i) changes in inequality in the determinants of health care use; and ii) changes in the elasticities of the correspondent determinants by cross-sectional unit or over time [1].

In addition, we used the bootstrapping method with 1000 replications to obtain confidence interval for the concentration index and horizontal index. We performed statistical analysis with the STATA MP 12.1 (StataCorp LP, TEXAS).

## Results

### Descriptive statistics

The descriptive statistics for all variables by study years are presented in Table 2. Some changes in primary, secondary and tertiary level health care use in outpatient visits were observed across the study years, albeit statistically insignificant. Overall inpatient utilization (hospitalization) and private hospital outpatient visits increased significantly from 2007/2008 to 2012. The results demonstrated that the SHI coverage increased these years and the increase was statistically significant.

**Table 2:** Descriptive statistics

Variables	2007/2008 (*n* = 27,681)	2012 (*n* = 30,567)
	Percent	
Health		
Chronic disease	17.8 %	NA
Disability	5.3 %	5.5 %
The number of work/school days. median. min and max^a^	0 (0, 31)	0 (0, 31)
Any health problem in last month	7.5 %	7.3 %
Age & sex		
Female 18–24^a,b^	12.0 %	10.5 %
Female 25–34	12.7 %	12.4 %
Female 35–44	12.2 %	11.7 %
Female 45–54^a^	8.5 %	9.6 %
Female 55–64^a^	4.0 %	4.8 %
Female 65–74	2.5 %	2.4 %
Female 74<	1.4 %	1.5 %
Male 18–24^a^	11.2 %	9.9 %
Male 25–34	11.4 %	11.6 %
Male 35–44	10.5 %	10.5 %
Male 45–54^a^	7.4 %	8.4 %
Male 55–64^a^	3.2 %	3.9 %
Male 65–74^a^	2.1 %	1.9 %
Male 74<^a^	0.8 %	0.9 %
Log income per capita. median. min and max^a^	13.9 (6.9, 19.4)	14.9 (11.9, 21.4)
Insurance coverage^a^	89.6 %	90.6 %
Household size. median (min. max)^a^	4 (1, 17)	4 (1, 15)
Marital status		
Married/living together^a,b^	60.3 %	63.5 %
Divorced/separated	3.5 %	3.4 %
Widowed	8.6 %	8.4 %
Single/never married^a^	27.7 %	24.7 %
Activity status		
Employed^a,b^	29.5 %	38.0 %
Herder^a^	13.4 %	15.5 %
Self-employed^a^	21.1 %	7.6 %
Inactive^a^	21.0 %	29.9 %
Unemployed^a^	15.0 %	9.0 %
Education		
None or lower education^a,b^	14.9 %	12.3 %
Secondary education^a^	56.9 %	55.4 %
Vocational^a^	11.0 %	11.6 %
Higher education^a^	17.2 %	20.7 %
Location		
Urban^a,b^	57.8 %	56.8 %
Rural^a^	42.2 %	43.2 %
Health care utilization		
Tertiary level health care oupatient visit	1.4 %	1.5 %
Secondary level health care outpatient visit	2.0 %	1.8 %
FGP/*soum* hospitals’ outpatient visit	1.7 %	1.8 %
Private hospital outpatient visit^a^	0.4 %	0.7 %
Any hospitalization^a^	12.3 %	13.3 %

^a^Statistically significant difference (*p* < 0.05) between 2007/2008 and 2012

^b^Reference group

### Total inequality and horizontal inequity

Table 3 summarizes the EI and horizontal inequity using equations 1–5.

**Table 3:** Erreygers’ concentration index and horizontal inequity by years

Health care utilization	2007/2008		2012	
	EI	HI	EI	HI
Tertiary level hospital outpatient visit (confidence interval)	**0.0056**	**0.0055**	**0.0078**	**0.0077**
	(0.0019, 0.0092)	(0.0019, 0.0089)	(0.0034, 0.0121)	(0.0040, 0.0111)
Secondary level hospital outpatient visit (confidence interval)	−0.0005	−0.0003	−**0.0050**	−**0.0054**
	(−0.0048, 0.0039)	(−0.0040, 0.0035)	(−0.0088, −0.0013)	(−0.0087, −0.0021)
FGP/*soum* hospitals’ outpatient care (confidence interval)	−**0.0052**	−**0.0053**	−**0.0082**	−**0.0088**
	(−0.0089, −0.0015)	(−0.0085, −0.0019)	(−0.0116, −0.0048)	(−0.0120, −0.0055)
Private hospital outpatient visit (confidence interval)	**0.0060**	**0.0060**	**0.0079**	**0.0074**
	(0.0037, 0.0082)	(0.0037, 0.0082)	(0.0050, 0.0106)	(0.0048, 0.0100)
Hospitalization (confidence interval)	**0.0159**	**0.0212**	**0.0169**	**0.0207**
	(0.0049, 0.0269)	(0.0110, 0.0312)	(0.0061, 0.0275)	(0.0099, 0.0294)

*EI* denotes Erreygers’ concentration index, *HI* represents horizontal Inequity. Significant indices are in bold, at the significance level of 0.01

*Tertiary level hospital outpatient visits*. The EI for tertiary level hospital outpatient visits in both years is positive and statistically significant at the level of 0.01. This shows that generally, higher income groups were more likely to utilize tertiary level outpatient care than lower income groups. After need variables were controlled, the horizontal inequity indices were positive and statistically significant in both study years. This explains that tertiary level outpatient care was distributed in favor of the pro-rich. Additionally, the EI and horizontal inequity indices substantially increased from 2007/2008 to 2012.

*Secondary level hospital outpatient visits*. The EI for 2012 was significantly negative, and this demonstrates that lower income group was more likely to use secondary level hospital outpatient care. The horizontal index for 2012 was −0.00544, indicating the pro-poor inequity; and secondary level of hospital outpatient care had a pro-poor distribution. The concentration indices and horizontal inequity indices were negative in 2007/2008; nevertheless, they were statistically insignificant. Therefore, in this paper, the results of the secondary level hospital outpatient visits were omitted from the subsequent analysis.

*FGP*/*soum hospitals*’ *outpatient visits*. The concentration indices were negative and statistically significant both in 2007/2008 and 2012. This result shows that lower income groups tend to seek FGP/*soum* hospitals’ outpatient care. Also, the horizontal inequity indices were negative in both years after standardization of need variables confirming that FGP/*soum* hospitals’ outpatient services were utilized more in favor of poor populations. The degree of inequality and inequity significantly increased from 2007/2008 to 2012.

*Private hospital outpatient visits*. In both the study years, the concentration indices and horizontal inequity indices for private hospital outpatient visits were significantly positive. In accordance with this result, the private hospital outpatient utilization distribution was generally concentrated among the rich, with significant inequity favoring the higher income groups. Furthermore, the degree of inequality and the degree of inequity rose during the study years from 0.0060 to 0.0079 and 0.0060 to 0.0074, respectively.

*Hospitalisation*. The concentration indices and horizontal inequity indices were positive and statistically significant for overall inpatient care, similar to the tertiary and private hospital outpatient visit results. This result indicates that hospitalization was more concentrated among the higher income patients and that the inpatient services were in favor of pro-rich. Also, the extent of inequality and inequity of the overall inpatient service distribution increased during the periods.

### Decomposition analysis

**Fig. 1:**
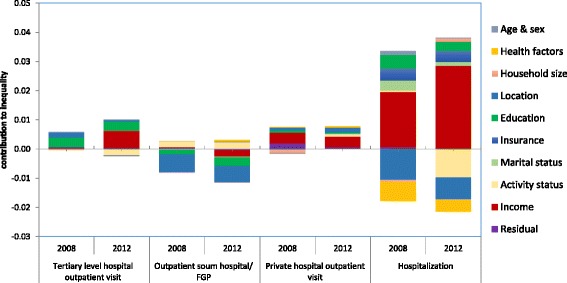
Decomposition analysis of inequalities in health care utilization, Mongolia, 2007/2008–2012

Figure 1 indicates the decomposition analysis for both years. We decomposed each inequality in health care utilization into need variables (age-sex dummies, health factors), non-need variables, and income. It enables us to observe which determinant contributes (its elasticity x its concentration index, see Additional file 1) more to inequality in health care utilization. If health care utilization is distributed equally across income, the total amount of the bars in the Fig. 1 is zero. On the other hand, if the health services are distributed equitably across income groups, the sum of these bars is equal to the need bar (sum of the health and age & sex), and it indicates the distribution of need across income. If there is a difference between actual and need adjusted distributions, the other bars appear in the figure. These bars denote the reasons for inequity.

The results demonstrate that the pro-rich inequity in tertiary level hospital outpatient use in both years was caused by income, education, and location. Income has largely increased from 2008 to 2012 the main contributors of inequity in tertiary level hospital outpatient use. The partial contributions of activity status and health factors were clear in the tertiary level hospital use inequity; nonetheless, the contributions were negative and comparatively small.

Similarly, pro-rich inequity was evident in private hospital outpatient use in both years. The major contributors to the inequity were income, location, education, activity status, and health factors. Interestingly, the contributions of income and education to inequity in private hospital outpatient use decreased whereas the contributions of other variables increased from 2007/2008 to 2012.

Furthermore, pro-rich inequity was obvious in inpatient service during both years. The substantial amount of contribution to inequity was from share of income, the SHI, marital status, and education. The positive contribution of the SHI to inequity in inpatient use can be explained by the health insurance co-payments.

Pro-poor inequity occurs in FGP/*soum* hospitals’ outpatient service in both years. This inequity was mainly driven by location, education, income, and activity status. In 2007/2008, the partial contribution of income to FGP/*soum* hospitals’ outpatient care use was positive; however, the contribution turned negative in 2012.

As seen in Fig. 1, income-related inequalities in health care utilization remarkably changed over time; however, what accounts for the change of each determinant of inequality in health care utilization lacks explanation.

Therefore, we conducted the Oaxaca-type decomposition because this helps us to decompose the changes of concentration indices and changes of elasticities by each determinant of health care utilization and shows us whether the change of a determinant is due to a change of the concentration index of a corresponding determinant or a change of elasticity in that determinant.

The summary of results is presented in the Additional file 2. Both equation 7 and 8 were used; however, a result of equation 8 is removed from the table due to limited space.

The total change in the concentration indices for tertiary level hospital outpatient visit, FGP/*soum* hospitals’ outpatient visit, private hospital outpatient visit, and inpatient service use were 0.0022, −0.0003, 0.0019, and 0.0009, respectively.

From 2007/2008 to 2012, income-related inequality increased in tertiary level hospital outpatient visits and private hospital outpatient visits; inpatient service use had a higher income group concentration. Income was the most influential determinant of increased inequality, and a change of concentration index of income was more important than a change of elasticities of income to contribute to such an increase of inequality. Impact of other determinants on increasing inequality was comparatively small.

During the study years, the income-related inequality of FGP/*soum* hospitals’ outpatient care increased among lower income populations due to a negative contribution of income. The negative contribution of income was caused more by change in the concentration index of income than it’s elasticities of income.

## Discussion

This study has produced some interesting findings. First, degrees of inequities in health service utilization have increased over time. In tertiary level hospital outpatient visits, private hospital outpatient visits and inpatient use concentrating on pro-rich populations. In terms of FGP/*soum* hospitals’ outpatient care, a pro-poor inequity in 2007/2007 was observed to have risen by 2012.

Second, while the poor have greater need, the rich use more health care services, except for FGP/*soum* hospitals’ outpatient services. FGP/*soum* hospitals’ outpatient care was higher among the poor proportionate to their needs. It confirms the results of a study conducted by the World Bank [22]; however, that study estimated only inequality in health care utilization and did not report about inequity in health care utilization. Furthermore, according to the MoH and the ADB report in 2010, the poor tend to visit primary health care more than the rich [35]. This can be explained by government policy to ensure that everyone has free access to primary health care which is fully funded by the state budget of Mongolia. Third, income contributed most to the pro-rich distribution in use of inpatient services. The previous study reported that inpatient services are expensive in terms of direct payment by user, transportation costs and other expenditures [36]. In addition, Nanzad et al. found that about 85 % of inpatients receive meals from their home every day and about 40 % inpatients purchase drugs and injections while hospitalizing at secondary level hospitals [37]. This is more burden for the lower income and vulnerable groups, as well as patients from rural areas. Similar findings were observed in the developing countries [38].

Other important factors which contributed largely to inequality in inpatient services are education, activity status, and insurance status. The positive contribution of insurance might due to the 10 and 15 % co-payment requirement for secondary and tertiary level hospitals, respectively. These co-payments led to lower hospital admission for the lower income groups owing to their lack of affordability. In terms of tertiary level hospital outpatient visits, location was one of the main contributors to the pro-rich distribution besides income and education. A possible explanation of the contribution of location is that all tertiary level hospitals are located in the capital city.

It is evident that income made the largest contribution to the pro-rich inequity in private outpatient visits. The rich tended to visit private hospitals due to factors such as short waiting time, pleasant environment, and among others. In addition, in recent decades, the number of private clinics and hospitals has increased considerably. Nonetheless, a great proportion of health care services burden is imposed on public services [27].

Likewise, there are several important findings for policy discussions. We may emphasize two of them which might be more urgent to address in the near future. One is about effective health service referral including the both public and private sector built on strong primary health care. This is essential for rational use of health services as well as containing health care cost in countries like Mongolia. Above findings indicate that the rich often bypasses cost effective primary health care and use more costly services at higher referral levels or in the poorly regulated private sector. This would lead to the future cost escalation, resource waste and inefficiencies in the health system, unless addressed properly. Another issue is financial access and risks protection. Currently, almost all Mongolian people have health insurance coverage on a mandatory basis regardless of their socio-economic characteristics. They all equally entitled to a set of defined health service benefits. The above findings show that health insurance coverage can be further analysed, discussed and improved in terms of effective coverage to ensure that all insured equally access and use of insurance benefits when they need them. This will reduce the gap that exists between legal and effective coverage thus the insured low income population who has greater need will have the same access and use of needed and quality health care at secondary and tertiary hospital levels. This will be an important policy issue for Mongolia where OOP has been increasing rapidly with limited share of health insurance in total health expenditure and high poverty rate referring to every third person. We think that followed discussions and policy actions to increase the share of prepaid financing arrangements including health insurance to reduce financial barrier in accessing health care as well as improve financial risk protection to prevent people from catastrophic health expenditure with impoverishing effects will be the main strategic direction for Mongolia to make rapid progress towards universal health coverage.

Van Doorslaer et al. conducted a research on income related-inequalities in doctor utilization among 12 European countries. They found that the degree of horizontal index of general practitioner visits in all 12 countries by probability was very small, a range between −0.016 and 0.012 [3]. The inequity degrees in FGP/*soum* hospital outpatient visits in Mongolia between 2007/2008 and 2012 increased as similar to those in Germany (0.008). However, pro-poor degrees in Mongolia is more concentrated than in 10 other European countries, comparing to results in Van Doorslaer et al. study results [3].

Van Doorslaer et al. also analysed equity in health services utilization, including general practitioners, specialists, and inpatient services in Europe and the US. For inpatient services, in 11 out of 12 countries, pro-poor inequity was observed, and much wider confidence intervals for those inequity indices were reported [2]. However, our study results demonstrated that inpatient services were concentrated among the rich even after need standardization, and the degree of inequity was relatively small.

In higher income Asian countries, Lu reported that pro-poor inequity in inpatient services was observed in South Korea (−0.0627), and Taiwan (−0.038) while pro-rich inequity was reported in Hong Kong (0.0638). The same study found that general practitioners’ visits were more concentrated among the poor, and pro-poor inequity was reported [4].

Comparing with developing countries, horizontal inequity in hospitalization in Mongolia was more equitable than those in Mexico (0.0269); however, it was more pro-rich than it was in Chile (0.015) [39, 40]. While, pro-poor inequity in hospitalization (−0.0127) was reported in Brazil [6].

As we believe that this study has the following strengths. We used a comparatively new method, the EI, which was developed as a solution for the limitation of standard concentration index. Further, we evaluated horizontal inequity, because inequality does not represent inequity in health care utilization. Moreover, we used income as the living standard measurement in the assessment; thus the different results from the studies with similar purposes can be explained.

This study has some limitations. The major one is that the HSES was designed to measure poverty and assess the living standards of the population. Thus, there was less information on health status and health care utilization compared to the Demographic and Health Surveys, used in similar studies in developed countries.

Specifically, there was no question for health behaviours or life style of individuals such as, physical activity, smoking, alcohol consumption, etc. On the other hand, the data did not allow us to capture the relationship between individuals’ income difference and quality of health service they received. Additionally, we were not able to measure the difference in health outcome among different income groups in the population. Some evidence depicted that income difference had impact on health outcomes of the patients. For instance, Canadian study results found that income level was significant and positively associated with the rate of coronary angiography and revascularization, while it was negatively correlated with waiting times of the same services. Most importantly, death rate within one-year after the procedures was significantly and negatively associated with income level [41].

Secondly, since the decomposition analysis is a descriptive statistic, we were not able to carry out a causality analysis. Thirdly, we analysed overall inpatient use; thus, there is a need to estimate horizontal inequity in the use of inpatient services by type of hospital.

## Conclusions

Equitable distribution of health care has been well documented in health strategies and policies in Mongolia, but the degree of inequity in delivery of health services has tended to increase. This does not directly imply that the degree of inequality in health has also increased at the same time. The implication of strategies and policy prioritizations need to be reconsidered. It is necessary to strengthen primary health care services, particularly by diminishing obstacles for lower income and higher need groups.

## Additional files

Additional file 1:
**Inequality decomposition for health care utilization, 2007/2008 and 2012.**
Additional file 2:
**Oaxaca-type decomposition of change in concentration index for health utilization variables, Mongolia, 2007/2008–2012.**

## Abbreviations

SHI: Social health insurance
OOP: Out of pocket payment
HSES: Mongolian Household Socio-Economic Survey
FGP: Family group practice
GDP: Gross domestic product
WHO: World Health Organization
EI: Erreygers concentration index
OLS: Ordinary least square
OECD: The Organization for Economic Cooperation and Development
MoH: Ministry of Health
ADB: Asian Development Bank
US: United States
